# Influence of minimally invasive cavities on color stability of dental crowns with different filling sealers

**DOI:** 10.1590/1807-3107bor-2024.vol38.0104

**Published:** 2024-11-08

**Authors:** Alice Corrêa Silva-Sousa, Manoel Damião Sousa-Neto, Rafael Verardino Camargo, Thamires Diogo Lima, Antônio Castelo Branco, Fernanda de Carvalho Panzeri Pires-De-Souza, André Luís Faria-E-Silva, Francisco Wanderley Garcia Paula-Silva, Renato Roperto, Aline Evangelista Souza-Gabriel, Jardel Francisco Mazzi-Chaves

**Affiliations:** (a) Universidade de São Paulo – USP, School of Dentistry of Ribeirão Preto, Department of Restorative Dentistry, Ribeirão Preto, SP, Brazil.; (b) Universidade de São Paulo – USP, School of Dentistry of Ribeirão Preto, Department of Dental Materials and Prosthodontics, Ribeirão Preto, SP, Brazil.; (c) Universidade Federal de Sergipe – UFSE, Department of Dentistry, Aracaju, SE, Brazil.; (d) Universidade de São Paulo – USP, School of Dentistry of Ribeirão Preto, Department of Pediatrics, Ribeirão Preto, SP, Brazil.; (e) University of Nebraska Medical Center – UNMC, College of Dentistry, Adult Restorative Dentistry Department, Lincoln, NE, USA.

**Keywords:** X-Ray Microtomography, Dental Pulp Cavity, Root Canal Obturation

## Abstract

The minimally invasive endodontic access is not directly associated with tooth discoloration in the presence of bioceramic or epoxy resin-based root canal sealers. This study aimed to evaluate the influence of minimally invasive access and endodontic sealer composition on the color stability of endodontically-treated teeth, the restorative material adaptation, and the presence of remaining filling material in the pulp chamber. Endodontic access surgery was performed in maxillary central incisors, either through conservative or minimally invasive approaches, and the root was filled with AH Plus or Bio-C Sealer. The crown color was measured with a spectrophotometer at baseline and after root obturation, restoration, and specimen storage for one year in an oven. The occurrence of voids in the restoration and the remaining filling material was analyzed using micro-CT scans. The Yellowness Index (YI) and color changes (∆E_00_) were calculated after each color measurement. Data of micro-CT were submitted to 2-way ANOVA, and YI and ∆E_00_ were analyzed with repeated-measures ANOVA. Pair-wise comparisons were performed with Tukey's test (α = 0.05). The experimental conditions had no effect on the presence of the remaining material. The minimally invasive access associated with Bio-C Sealer resulted in more voids between the restoration and the remaining filling material. Only the evaluation time affected YI and ∆E_00_ values. Specimens became more yellow after filling and storage in the oven (the highest ∆E_00_ values). The present study showed that sealer and minimally invasive cavities are not associated with crown color stability following endodontic treatment.

## Introduction

Minimally invasive endodontic cavities preserving part of the pulp chamber roof and pericervical dentin have been proposed to conserve the remaining tooth structure and increase its fracture resistance after performing definitive restorative procedures on teeth.^1–4^ This approach involves creating smaller access cavities that avoid extensive removal of the tooth's structural components. However, preserving parts of the pulp chamber roof may prevent the complete removal of pulpal tissue and facilitate the retention of staining substances from antibiotic pastes, intracanal medications, root canal sealers, and irrigating solutions.^5,6^

Color changes in endodontically treated teeth can be associated with the presence of endodontic sealers containing silver and bismuth oxides, as radiopacifying agents, in the pulp chamber.^7,8^ The color of bismuth oxide changes from yellow to dark brown in the presence of irrigating solutions (*e.g.*, sodium hypochlorite), collagen from remaining dentin, blood, and lack of free oxygen.^8–10^ Therefore, radiopacifiers based on silver oxide and bismuth oxide have been replaced by zirconium oxide and iron (*e.g.*, AH Plus),^10,11^ and bioceramic sealers were developed.^7,8^ Epoxy resin-based sealers may lead to, however, progressive coronal discoloration over time.^9^ On the other hand, only imperceptible discoloration has been reported with bioceramic sealers.^12,13^

Color evaluations with spectrophotometers are commonly employed to reduce possible biases related to subjective analysis. The Commission Internationale l'Eclariage (CIE) has defined systems to quantify color using coordinates indicating lightness (L* or Y) and chromaticity. The CIELAB system quantifies the chromaticity through coordinates a* (red-green axis) and b* (yellow-blue axis). Color differences are usually measured using either the CIE76 (∆E_ab_) or CIEDE2000 (∆E_00_) formulas.^14^ Although ∆E values are commonly used in dental studies, conclusions based only on these data are problematic. For instance, they do not indicate whether the specimens became yellower or whiter. Thus, using the yellowness index can help to better understand the color changes observed after endodontic treatment.^15,16^

Tooth discoloration can also be due to voids around the restoration and remnants of filling material (*i.e.* sealer and gutta-percha). Micro-computed tomography (micro-CT) is a useful tool to evaluate the volume of remaining sealer and voids in the restoration.^17–20^ Therefore, this study evaluated the impact of the type of access surgery on the color stability of endodontically treated teeth obturated with either an epoxy resin-based sealer or a bioceramic sealer. Moreover, the remaining filling material in the pulp chamber and voids in the restoration were also assessed. The null hypothesis of this study is that neither: a) the endodontic access approach or b) the sealer composition affects the tooth color.

## Methods

This *in vitro* study was approved by the Research Ethics Committee (CAAE: 40959720.0.000.5419) of the Faculty of Dentistry of Ribeirão Preto, University of São Paulo. Fifty sound human maxillary central incisors were scanned in a high-resolution micro-CT device (SkyScan 1174, Bruker, Kontich, Belgium), and 32 were selected based on the similarity of enamel (0.7–1.0 mm) and dentin (2.0–2.5 mm) thicknesses. The sample size was calculated for the continuous outcome ∆E_00_, defined as primary. A pilot study was carried out and showed a standard deviation of 1.13 (mean 2.41) in ∆E_00_ values due to root canal obturation. Therefore, considering the threshold of 1.8 as clinically acceptable,^21^ the Cohen's D effect size calculated was 1.59. The sample size calculation was performed for a two-sided T-test assuming a type-I error of 5% and a test power of 80%. The G*Power software (version 3.1.9.6; Duesseldorf, Germany) was used for the calculation, and at least eight specimens per experimental condition were defined as necessary to meet these parameters. All selected teeth presented complete rhizogenesis, a single root canal, mild root curvature (°r > 8 mm),^22^ and no calcifications or resorptions.

The initial tooth color was recorded on the buccal surface with a spectrophotometer (Vita Easyshade V, VITA Zahnfabrik, Bad Säckinge, Germany) based on the CIELAB system. An individualized silicone index was used to standardize the color reading area for the entire experiment, and readings were performed in triplicate. A tool available on the website Nix™ Color Sensor (https://www.nixsensor.com/free-color-converter) was used to convert the color coordinates from the CIELAB system to the CIEXYZ. The conversion considered a 2° observer and D65 illuminant, the defaults of the spectrophotometer used. YI of specimens was calculated using the ASTM Method E313:


$$YI=\frac{100\times \left(C_{x}\times X–C_{z}\times Z\right)}{Y}$$

Where C_x_ (1.2985) and C_z_ (1.1335) are the coefficients for the X and Z coordinates, respectively, for the observer angle and illuminant used in the present study.

Specimens were also scanned with the high-resolution micro-CT scanner. Geometric and flat-field corrections were obtained by positioning a 0.5-mm aluminum filter between the specimens and the X-ray source, altering the sensitivity to polychromatic radiation and reducing the possible hardening effect. A polystyrene tube was used to keep specimens perpendicular to the radiation source and reduce possible image distortions. Specimens were scanned under 50 kV, 800 mA, isotropic resolution of 26.7 mm, and 360° rotation. Images were reconstructed using the NRecon v.1.6.3 software (Bruker, Kontich, Belgium) and CTAN v.1.15.4 software (Bruker, Kontich, Belgium).

A single experienced endodontist treated and restored all the canals using an operating microscope. The access to the pulp chamber for the conservative endodontic cavity (CEC) was carried out with high-speed rotating spheric diamond burs (#1013 and 1014, KG Sorensen, São Paulo, Brazil), followed by EndoZ burs (KG Sorensen, São Paulo, Brazil). Only the spheric diamond burs 1011HL (KG Sorensen, São Paulo, Brazil) were used for the minimally invasive endodontic cavity (MEC) (n = 16). After that, the root canal was explored with a #15 K-type hand file (Dentsply Maillefer, Baillagues, Switzerland) until its tip was visible at the apical foramen and the working length (WL) was set 0.5 mm shorter than this measurement. Then, following the manufacturer's instructions, the patency of the root canal was performed with the WaveOne Gold Glider (#15) instrument (Dentsply Maillefer, Baillagues, Switzerland), and the anatomical diameter was determined with a #20 K-type hand file (Dentsply Maillefer, Baillagues, Switzerland) at the WL.

The biomechanical instrumentation was performed with a single WaveOne Gold Large (#45) instrument (Dentsply Maillefer, Baillagues, Switzerland), under a reciprocating movement, following the manufacturer's directions. After three pecking motions, the instrument was removed, and the root canals were irrigated with 5 mL of 2.5% sodium hypochlorite^23^. Root canals were irrigated with 2 mL of 17% EDTA for 5 minutes, dried with absorbent paper points, and filled with a single gutta-percha cone corresponding to the final diameter #45 (Conform fit, Dentsply Maillefer, Baillagues, Switzerland). The obturation was performed using the single cone technique. Before its insertion, the cone was covered with either (AH Plus Dentsply, De Trey, Konstanz, Germany) or Bio-C Sealer (Angelus, Londrina, PR, Brazil) (n = 8). The excess obturating material was removed with a heated instrument 2 mm below the cementoenamel junction. Vertical condensation of plasticized gutta-percha was performed using Paiva condensers. Finally, the endodontic cavity was cleaned with sterile cotton soaked in 70% alcohol and an ultrasonic tip without refrigeration (E1 – Irrisonic Tip, Helse, Santa Rosa do Viterbo, SP, Brazil).

The tooth color was measured again, and YI was calculated using previously described procedures. Data from CIELab were converted into CIELCh, and the overall color change caused by the obturation was calculated using the following formula (CIEDE2000):


∆E00=(∆L'KLSL)2+(∆C'KCSC)2+(∆H'KHSH)2+RT∆C'KCSC∆H'KHSH'

Changes in lightness (∆L’), chroma (∆C’), and hue (∆H’) were calculated by subtracting the value measured from the values of the prior step. S_L_, S_C_, and S_H_ are the weighted functions for each component. K_L_, K_C_, and K_H_ are the weighted factors for lightness, chroma, and hue, respectively (it was used K_L_ = K_C_ = K_H_ = 1). R_T_ is the interactive term between chroma and hue differences.

Immediately after the obturation, the enamel surrounding the cavity was etched with 37% phosphoric acid (Dentsply Maillefer, Ballaigues, Switzerland) for 30s, rinsed with water and dried. A universal adhesive (Single Bond Universal, 3M ESPE, St. Paul, USA) was applied on the cavity with vigorous rubbing for 15 s and light-cured for 20 s. Cavities were incrementally restored with a nanofiller resin composite (Filtek Z-350 XT, shade A2, 3M ESPE, St. Paul, USA). Each increment was cured for 20 s with a LED-based light-curing unit (Radii Plus, SDI, Limited, Bayswater, Victoria, Australia) with an irradiance of 1,500 mW/cm^2^. Specimens were stored at 37°C under absolute humidity for 24h.

The tooth color was measured again, and YI and ∆E_00_ resulting from the restorative procedure were calculated. Specimens were also scanned, and images were reconstructed with the same parameters as the initial scan. The occurrence of voids around the restoration and remnants of filling material (sealer and gutta-percha) in the tooth crown was analyzed. The bottom of the section was considered the cementoenamel junction and the top, the incisal edge. Then, the voids were identified using the threshold scale in the CTAn software (Bruker, Kontich, Belgium). The "3D analysis tool" automatically calculated the volume of voids, considering the selected threshold^20^. The same procedure was carried out to calculate the remnants of filling material in the pulp chamber. Three-dimensional images were used for qualitative analysis using the Data Viewer v.1.5.1 64-bit (Bruker, Kontich, Belgium) and CTVol v.2.3.1 (Bruker, Kontich, Belgium) software. The specimens were stored in distilled water (replaced every day) for one year in an oven at 37°C.

Data of each outcome were individually tested regarding the normal distribution (Shapiro-Wilk test) and sphericity (Mauchly's W, Greenhouse-Geisser, and Huynh-Feldt tests). Two-way ANOVA ("sealer" vs. "endodontic access") was used to analyze data for voids in restorations and remnants of filling material in the pulpal chamber. For ∆E_00_ and YI, statistical analysis was carried out using repeated-measured (RM) ANOVA to evaluate the independent variables "sealer", "endodontic access", and "assessment time". The last variable was defined as a factor for repeated measures. Pair-wise comparisons were performed using Tukey's test. A confidence level of 95% was pre-set for all analyses, which were carried out using the open statistical platform Jamovi 1.6.15 (www.jamovi.org).

## Results

### Micro-CT analysis

**Figure 1:**
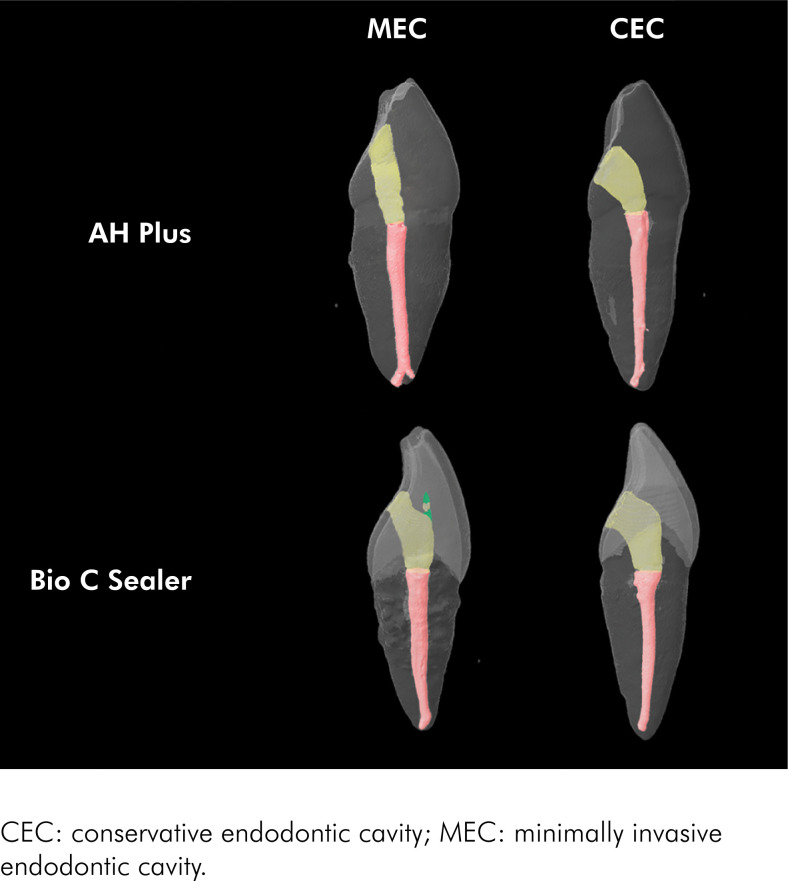
Representative images reconstructed from micro-CT scans according to endodontic access and sealer. Note the presence of voids (green) for MEC and Bio-C Sealer.

Neither "sealer" (p = 0.218) nor "endodontic access" (p = 0.522) affected the presence of filling material in the pulpal chamber, and the interaction between these factors was also not significant (p = 0.399) (Table 1). Two-way ANOVA showed that both independent variables (sealer, p = 0.077; endodontic access, p = 0.848) had no significant effect on the occurrence of voids in restorations. However, the interaction between the variables was significant (p = 0.022) (Table 1). Differences between sealers were observed only with MEC. Root canals accessed using MEC and filled with Bio-C Sealer resulted in more voids around the crown restoration compared to using AH Plus. The endodontic access did not affect the presence of voids when AH Plus was used but using Bio-C Sealer resulted in more voids for MEC than CEC. Figure 1 shows representative images reconstructed using micro-CT scans. It is possible to observe the presence of voids in green for Bio-C Sealer and MEC.

**Table 1:** Means (standard deviations) of filling material residues and voids according to endodontic accesses and sealers (n = 8).

		Remaining filling material		Voids	
Variable		AH Plus	Bio C Sealer	AH Plus	Bio C Sealer
		Mean (SD)	Mean (SD)	Mean (SD)	Mean (SD)
Endodontic access					
	CEC	0.08 (0.17) ^Aa^	0.01 (0.03) ^Aa^	1.16 (1.18) ^Aa^	0.99 (0.41) ^Aa^
	MEC	0.03 (0.04) ^Aa^	0.02 (0.02) ^Aa^	0.92 (0.43) ^Ab^	1.75 (0.93) ^Aa^

For each outcome, distinct letters (uppercase comparing endodontic accesses; lowercase comparing sealers) indicate statistical difference with Tukey's test (p < 0.05). CEC: conservative endodontic cavity; MEC: minimally invasive endodontic cavity.

### Yellowness index

**Figure 2:**
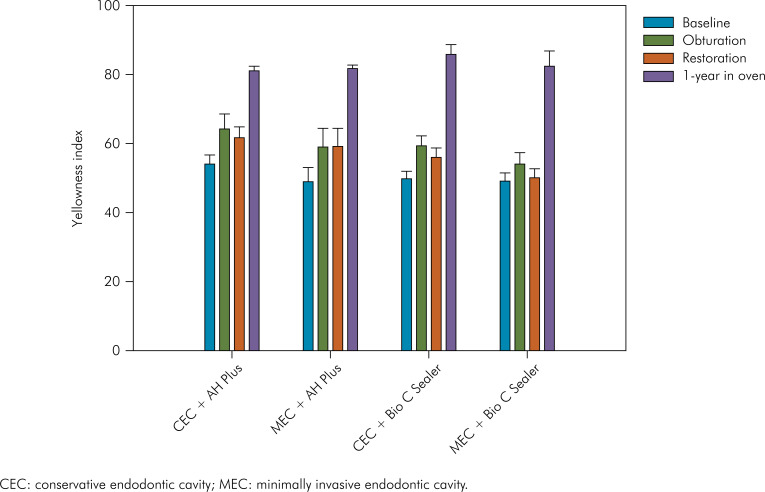
Bar graph showing means and standard errors of yellowness index according to the experimental conditions.

Results for YI are presented in Figure 2. RM ANOVA showed that only the variable "assessment time" (p < .001) affected the values of YI. Neither "sealer" (p = 0.307) nor "endodontic access" (p = 0.227) had a significant effect on this outcome. Only the double interaction "sealer vs. assessment time" (p = 0.021) was significant. The p-values for other interactions were: "sealer vs. endodontic access", p = 0.887; "endodontic access vs. assessment time", p = 0.666), and the triple interaction (p = 0.560). Table 2 presents the results of Tukey's test for the interaction between the variables "assessment time" and "sealer". Regardless of the endodontic sealer, the specimens were yellower after the obturation than at baseline. The restoration slightly reduced the yellowness but without difference to the values observed after obturation. The difference between restored specimens and baseline was maintained only for the AH Plus. For both sealers, the highest values of YI were observed after the 1-year storage in the oven. No difference between the sealers was observed for all assessment times.

**Table 2:** Means (standard deviations) of yellowness index according to sealers and assessment times (n = 16).

	Sealer	
Assessment time	AH Plus	Bio C Sealer
	Mean (SD)	Mean (SD)
Baseline	51.4 (9.9) ^Ca^	49.5 (5.4) ^Ca^
Obturation	61.6 (13.7) ^Ba^	56.7 (9.0) ^Ba^
Restoration	60.4 (12.2)^Ba^	53.1 (7.7)^BCa^
1-year storage in oven	81.4 (3.1) ^Aa^	84.1 (10.6) ^Aa^

For each outcome, distinct letters (uppercase comparing assessment times; lowercase comparing sealers) indicate statistical difference with Tukey's test (p < 0.05).

### Color changes after each step

**Figure 3:**
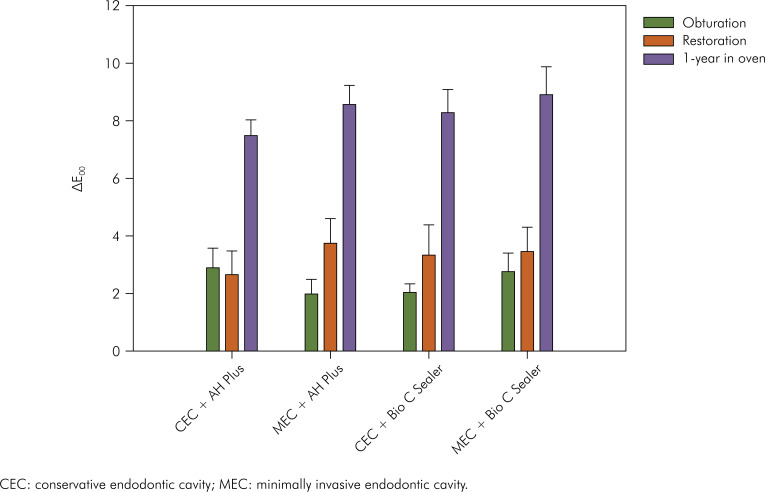
Bar graph showing means and standard errors of ∆E_00_ according to the experimental conditions.

**Figure 4:**
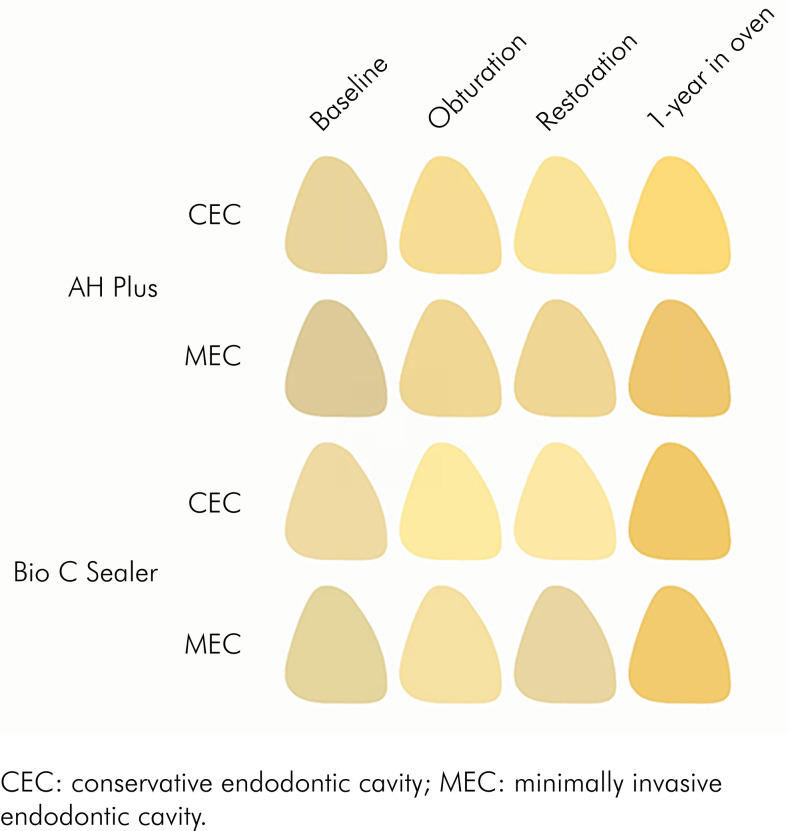
Illustrative image of color changes observed after each step according to endodontic access and sealer.

Results for ∆E_00_ are displayed in Figure 3. RM ANOVA showed that only the variable "assessment time" (p < 0.001) affected the values of ∆E_00_. Neither "sealer" (p = 0.537) nor "endodontic access" (p = 0.239) had a significant effect on this outcome. Moreover, all double interactions ("sealer vs. endodontic access", p = 0.908; "sealer vs. assessment time", p = 0.830; and "endodontic access vs. assessment time", p = 0.609), and the triple interaction (p = 0.391) were not significant. Pooled averages for the variable "assessment time" are presented in Table 3. Similar color changes were caused by both obturation and restoration, and the highest values of ∆E00 were observed after 1-year storage in the oven. Color coordinates were used to draw Figure 4, which illustrates the color changes caused by each step according to the experimental conditions.

**Table 3:** Means (standard deviations) of ∆E_00_ observed according to assessment times (n = 32).

Assessment time	Outcome
	∆E_00_
Obturation	2.4 (1.6) ^B^
Restoration	3.2 (1.2) ^B^
1-year in oven	8.3 (2.1) ^A^

For each outcome, distinct letters indicate statistical difference with Tukey's test (p < 0.05).

## Discussion

This study's results confirmed the hypotheses that neither endodontic access approach nor sealer composition affect tooth color. Tooth discoloration (darkening and yellowing) after endodontic treatment can result in patient dissatisfaction with the treatment in esthetic areas, negatively impacting quality of life.^24,25^ Therefore, establishing clinical protocols and materials that prevent significant and undesirable tooth color changes might be as important to treatment success as eliminating the infection in root canals. A factor associated with tooth discoloration is residual filling material (*i.e.*, sealers and gutta-percha) in the pulpal chamber. Hence, in the present study, to evaluate the influence of type of cavity and filling material on the color stability of the crown, other variables, such as saliva and food residue, were considered constant.

Some studies have suggested that using MEC can result in more filling material remnants in the pulp chamber, including areas below the pulpal chamber roof.^23,26^ However, the micro-CT analysis did not demonstrate any significant effect of the endodontic access protocols evaluated in the present study on the volume of remaining filling material in the pulpal chamber. Moreover, the sealer used had no effect. The use of assistive technologies, such as magnification by operating microscope and ultrasound, along with the operator's experience in the present study, facilitates the complete removal of the filling material and can explain the results.^3,27^ It is noteworthy that the operating microscopy improves visualization and allows the operator to access difficult areas with the ultrasonic insert, which breaks and extracts the filling material by acoustic transmission.^28,29^

On the other hand, the highest occurrence of voids surrounding the restoration was observed for the obturation using Bio-C Sealer in MEC. Bio-C Sealer contains high free CaO content, low concentrations of C_3_S (tricalcium silicate) and C_2_S (dicalcium silicate), and a long-chain polymer. This composition favors crystal formation by hydration reactions, which impairs crystal interlacing.^29,30^ This disrupted crystal structure in Bio-C Sealer could explain the higher number of voids observed, which might weaken the bond between the composite filling and the dentin walls of the cavity. This weakening effect could be more pronounced with higher shrinkage stress in the composite. With MEC, the ratio between bonded and non-bonded areas of the restoration (the so-called C-factor) tends to be higher than with CEC. Consequently, higher shrinkage stress of the composite is expected, leading to a more pronounced effect of the presence of Bio-C Sealer. While this explanation is a possibility, additional studies are needed to definitively confirm the link between Bio-C Sealer and increased voids, particularly in the context of MEC.

Regarding tooth discoloration, the present findings showed that the obturation of the root canal caused the specimen's zx yellowing. For specimens filled with Bio-C Sealer, however, the tooth color after restoring the endodontic access cavities was similar to that measured at baseline. Besides, the 1-year storage resulted in the most severe yellowing for both sealers. For the epoxy resin-based sealer AH Plus, tooth yellowing may be related to its chemical interaction with the dentin collagen matrix.^6^ The high flowability of this sealer improves its penetration into the dentinal tubules favoring some color change of the dentin.^31^ It is also worth noting that thermoset polymers, including epoxy resins, are susceptible to physical aging over time.^16^ Physical aging relies on changes in polymer structure towards a thermodynamic equilibrium, which is ultimately followed by a polymer color change.^32^ Moreover, the spatial geometric configuration of the epoxy molecule is prone to water absorption due to its open structure and the presence of cross-linking agents.^33^ Therefore, a gradual color change can be expected due to the degradation of curing initiators over time.

Regarding the Bio-C Sealer, the discoloration mechanism is associated with sealer oxidation. During its setting, the calcium ion released by the hydration reaction reacts with phosphate ions and plasma proteins from the dentinal fluid, resulting in pigmented subproducts, such as calcium phosphate.^31^ In addition, the iron oxide from the radiopacifying agent is reduced to aluminoferrite during the hydration process of bioceramic sealers, modifying the color of the sealer from white to yellow-grey.^34^ Finally, as the sealer sets, hydrated compounds such as hydrated calcium silicates (an amorphous structure) and calcium hydroxide are formed, which crystallize in superposed hexagonal plates. As the sealer becomes more porous and chemically reactive, it can absorb higher content of organic and inorganic components (e.g., necrotic tissue, smear layer, and blood cells), favoring color changes over time.^35^ It is important to emphasize that both sealers contain zirconium oxide as a radiopacifying agent that degrades in the presence of sodium hypochlorite and EDTA remaining in the dentinal tubules.^14,36–40^ The degradation of this agent also contributes to tooth discoloration.

The findings of this study highlight the clinical relevance of minimally invasive endodontic access, demonstrating that it is not directly associated with tooth discoloration when using bioceramic or epoxy resin-based root canal sealers. This suggests that dentists can adopt minimally invasive techniques without increased risk of esthetic compromise due to discoloration. However, it is important to acknowledge the limitations of this study, as it was conducted in vitro. The controlled environment of an in vitro study cannot fully replicate the complex conditions encountered in clinical practice, such as variations in oral flora, patient-specific factors, and long-term outcomes. Future research should aim to validate these results in clinical trials to better understand the implications of minimally invasive endodontic access in real-life scenarios.

Residues of filling materials on the dentin surface may lead to tooth crown darkening over time. This occurrence was not dependent on the root canal sealer composition (epoxy resin-based or bioceramic-based) and the type of endodontic access cavity (minimally invasive or conservative). The physicochemical changes in sealers are responsible for tooth discoloration, impacting the esthetics of the patient's smile. Thus, protocols to effectively remove the endodontic sealer remnants from the pulpal chamber should be proposed to minimize this clinical effect.

## Conclusions

A minimally invasive endodontic access does not affect the tooth color in the presence of either bioceramic or epoxy resin-based sealers. Irrespective of the sealer, the yellowing of specimens was observed after the obturation of the root canal. The restorative procedure only compensated this color change for specimens obturated with Bio-C Sealer. The more pronounced color changes occurred after a 1-year storage of the specimens in distilled water.
